# Correction: Acceptability of unsupervised peer-based distribution of HIV oral self-testing for the hard-to-reach in rural KwaZulu Natal, South Africa: Results from a demonstration study

**DOI:** 10.1371/journal.pone.0298143

**Published:** 2024-01-29

**Authors:** Marcel K. Kitenge, Chinmay Laxmeshwar, Elkin Bermudez Aza, Ellie Ford-Kamara, Gilles Van Cutsem, Ntombi Gcwensa, Esther C. Casas, Khanyo Hlophe, Petros Isaakidis, Liesbet Ohler

There are errors in the Funding and Competing Interests statements. The correct statements are:

**Funding:** Médecins sans Frontières provided funding for the study. All the authors except for KH are or were employed part-time or full-time by Médecins sans Frontières and received support in the form of salaries. The specific roles of these authors are articulated in the ‘author contributions’ section. Additionally, MSF supplied the oral self-tests for the study.

**Competing Interests:** The authors have read the journal’s policy and have the following competing interests to declare: MSF provided support for this study in the form of salaries for MKK, CL, EBA, EF, GVC, NG, ECC, PI, and LO. This does not alter our adherence to *PLOS ONE* policies on sharing data and materials. There are no patents, products in development or marketed products associated with this research to declare.
