# Supplementary material for: Acceptability of unsupervised peer-based distribution of HIV oral self-testing for the hard-to-reach in rural KwaZulu Natal, South Africa: Results from a demonstration study
Source: PLoS One. 2022 Mar 30;17(3):e0264442. doi: 10.1371/journal.pone.0264442 (PMC8967037; doi:10.1371/journal.pone.0264442)
Supplement: Attachment — Submitted filename: Responses to reviewers.docx [file pone.0264442.s001.docx]

24 December 2021

Dear reviewers,

Re: Responses to reviewers’ comments

Please find below a point-by-point response to the issues raised by the reviewers. The changes made to the manuscript are highlighted in track changes in the revised version.

**Original Title**: Feasibility and acceptability of unsupervised peer-based distribution of HIV oral self-testing for the hard-to-reach in rural KwaZulu Natal, South Africa: Results from a demonstration study .

# Reviewer #1

**Comment 1**

Background
line 84:More than two-thirds of people living with HIV (PLHIV) are in Africa'' it would great to make reference of the year.

**Response**

We thank the reviewer for the comment. We have added the year as shown in line 94 and tis read as follows “*In 2019, more than two-thirds of people living with HIV (PLHIV) are in Africa.*”

**Comment 2**

**Methods**
line 116: Expand your design section, read on scientific methodological design

Response

We appreciate the reviewer’s comment. We have expanded the study design as shown 135-139 and this reads as follows “*This was a serial cross-sectional study of peer-led, unsupervised distribution model of HIVST kits. Whereby, lay counsellors or community health workers provided HIVST kits to primary recipients (PRs) for distribution to their sexual partner, anyone in their social network (termed secondary recipients) or for self-testing.*”

**Comment 3**
line 148_149 ''Those diagnosed with HIV on the 148 day of visit were excluded'' what was the reason for excluding, would you agree that perhaps the partners of those whom tested positive might have needed the kits or missed due to this , and was everyone tested before handing out the HIVST kits.

**Response**

We thank the reviewer for the comment. We fully agree with reviewer assertion that excluding those whom tested positive may have missed an opportunity to get their partners tested. However, excluding these newly diagnosed with HIV on the day of enrolment was motivated by the fact that predominant majority of newly diagnosed patients are not always in right state of mind considering the adverse psychological burden they present as they just discovered to have a life changing disease. Asking these patients to consent in order to take part in the study would spare unnecessary emotional and psychological distress. Due to these reasons we decided to excluded them in the study.

We have added justification of this exclusion in line 184-186 and this reads as follows “*Given the emotional and psychological distress associated with a new HIV-positive diagnosis, those diagnosed with HIV on the day of visit were excluded.*”

**Comment 4**

**Passive follow up:**
why passive follow up?
What happened to those who reported back to not study/participating facilities. Were neighbouring facilities informed of the ongoing study and how to respond to HIVST bought back?

**Response**

We appreciate the reviewer’s comment. Why passive follow-up ? this paper is of particular significance considering it is demonstrating how HIVST peer-distribution strategy would work in the real-world conditions. Therefore, actively following primary recipients would have defeated the intended objective. We stated in line 200-203 that “*After handing over the tests to the PRs, we did not actively follow them up. HIVST users would decide on their own agency to visit the clinic for confirmatory testing and ART initiation*.”

Furthermore, the study settings consisted of 27 sites all sites (PHC and community-HIV testing sites). During the study period, everyone coming to the public health facilities and community HIV testing sites was asked if they had used an HIVST and from whom they received an HIVST. (in our catchment area) as stated in line 204-207 and this reads as follows “*During the study period, everyone coming to the study sites for HIV testing was asked if they had used HIVST and from whom they received an HIVST. In case they reported using HIVST, with or without presenting the care card, they were informed about the study and asked for consent to be enrolled in the study.*” However, some individuals may have reported outside of our catchment area or study setting.

**Comment 5**

Results :
line 221: 34,715 adults were tested for HIV at both PHCs and community-based testing sites, among whom 1,089 participants reported HIVST use'' so the 1089 already knew their HIV status before being handed the test kits?

**Response**

We thank the reviewer for the comment. As we the stated in previous response, during the study period, everyone coming to public health facilities in the catchment area was asked if they had used an HIVST and from whom they received an HIVST. During the study 34,715 adults were tested for HIV at both PHCs and community-based testing sites, therefore , out of 34,715 adults, 1089 reported having used an HIVST and had come for confirmatory testing. We have added in sentence as shown in line 207-209 and this read as follows “*Following the consent process, the study’s counsellor conducted individual face-to-face using SRs questionnaire, during which those HIVST users self-reported their HIVST results.* ”

**Comment**
line 223: the 196 individuals who tested positive, was this on site or during follow up, assuming everyone was tested, this means some of these ( PR's) were HIV negative at baseline? please clarify

**Response**

We appreciate the reviewer’s comment. All 196 received a confirmatory test on site during the follow following positive HIVS shown in line 310-312 and this reads as follows “*Of 196 (18%) who reported positive HIVST results and arrived at both PHCs and community-based testing sites for confirmatory testing, all reported having performed the HIVST within a median time two months (IQR: 1.15-2.85)”.* Additionally, our study did not seek to actively follow-up PRs (until they return to clinic or community HIV-testing for confirmatory test), we cannot confirm with certainty that some PRs had sero-converted during the study period. However, we have acknowledged this as one of the study limitations. See line 411-414 and this reads as follows “*An inherent limitation of many studies of unassisted self-testing is the difficulty of verifying use and accuracy of the tests. In our study all information in the follow-up component was obtained through participants’ reports, and thus we were unable to verify use, or results.”*

**Comment**
Figure 1: Only HIV positives reported, what happened to the other HIVST, are we assuming they were all negative, where there any unused kits returned to the facilities or study sites?

**Response**

We thank the reviewer for the comment. Figure 1 aimed to graphically present linkage to care among HIVST users and non-users. This is shown in 293-294 and it reads as follows “*Overall, 893 (82.0%) reported a negative HIVST, 196 (18.0%) reported a positive result and no participant reported an indeterminate result.”* Additionally , this information is also included in Table 3.

# Reviewer #2

**Comment**

Do not see the reason why ‘feasibility’ is included in this manuscript. Feasibility (feasible) is mentioned in the title, in the background (as related to other studies which looked at feasibility), in the first sentence of the Discussion section(as a finding from the study), and in the Conclusion (only in the Abstract section). There isn’t anything related to this in the methods or results. May need to remove this (feasibility/feasible) from the title and discussion and conclusion

**Response**

We appreciate the reviewer’s suggestion. We have removed the these words (feasibility/feasible) throughout the manuscript. See line 1 and 323.

**Comment**

Abstract: word count is 384 (vs 300 word limit for PLoS One). Many acronyms/ abbreviations are used (vs abstracts should not include Abbreviations, if possible, in PLoS ONE guideline)

**Response**

We appreciate the reviewer’s comment. We have reduced the word count and the current word count stand at 302 as shown in line 35-78.

**Comment**

Background: provides a good appraisal of what is known in the area and clearly highlights the rationale for conducting this study (and it is ‘acceptability’, nothing more mentioned here)

**Response**

We thank the reviewer for the comment. As highlighted in our first response. We have deleted ‘feasibility’ or ‘feasible’ as you suggested in your first comment.

**Comment**

Methods: from what is presented in this section, the study used a (prospective) cross-section design. One may even consider this as a cross-sectional analytical design, as clearly stated in the Analysis section (Line 184…) “Pearson’s Chi-squared and Fisher’s exact tests were used to assess the statistical significance of differences in categorical outcomes between selected groups. Two-tailed tests were used throughout, and the level of significance was set at 5%”. This means the one may describe this study as a cross-sectional analytical design (with prospective data collection). Also, a sample size is required for this study. Of note, it is also stated in Line 321 “The study was not powered to detect factors associated with linkage confirmatory testing and initiation of ART. What is power for? --- Sample Size? By just eyeballing, the sample seems adequate, but can one slip through without showing the sample size and the assumption involved in the estimation?

**Response**

We thank the reviewer for the comment and suggestions. We have changed the study design as shown in line 134-138 and this reads as follows “*This was an analytical cross-sectional study of peer-led, unsupervised distribution model of HIVST kits, whereby, lay counsellors or community health workers provided HIVST kits to primary recipients (PRs) for distribution to their sexual partner, anyone in their social network (termed secondary recipients) or for self-testing. In addition, data was collected prospectively among those returned for confirmatory testing”.*

Furthermore, in this study we distributed 31,341 HIVST kites through primary recipients in 17 month. One primary recipient could have received 2-5 HIVST kits for distribution and for use. Therefore , we did not have fixed sample size in terms of number of primary recipients to enrol in the study. We did have fixed number of HIVST kits to give out. In order to avoid confusion we have deleted line 321-324.

**Comment**

Results: Table captions should be complete to make them self-contained (add to the titles given place and date. It is helpful to provide expanded forms of unusual acronyms in the titles and bodies of tables as footnotes to the tables. Descriptions of some of the results provided in the tables may need to move to more appropriate sections/paragraphs to improve on the flow of content (comments given in sections of the results below). Use commas consistently in figures more than 4 digits, like 1,000 instead of 1000).

**Response**

We thank the reviewer for the comments and suggestions. We have expanded forms of unusual acronyms as shown in line 260-259, we have inserted commas in figures with more than 4 digits.

**Comment**

Specific comments:

Abstract:
Line 38: There is an extra space between the hyphen and distribution

**Response**

We appreciate the reviewer comment and this has been addressed as shown in line 38 and this reads as follows “*This study aimed to describe the acceptability of unsupervised peer-distribution of HIV self-testing as a method to scale-up HIV testing.”*

**Comment**
Line 42: May change partner to partners
**Response**

We thank the reviewer for the comment. This has addressed as shown in line 43 and this reads as follows *“…or community health workers provided HIVST kits to primary recipients (PRs) for distribution to their sexual partners,*”

**Comment**
Line 50: ‘accepted to be’, I think, should be changed to ‘were’

**Response**
We appreciate the reviewer’s comment. This has been addressed as shown in line 50 and this reads as follows “*Among 36,708 people approached to be primary recipients, 9,891 (26.9%) accepted*”
**Comment**

Line 52: ‘6,598 (66.7%)’ is out of place in the sentence; please edit.
**Response**

We appreciate the reviewer for the comment. “6,598 (66.7%)” has been deleted as show in line 52 and this reads as follows “*PRs were predominately recruited at primary health clinics (PHCs)*”

**Comment**
Line 58: I think, “who’ is missing between ‘64.7%)’ and ‘were”

Response
We thank the reviewer for the comment . The sentence has been changed as shown in line 56-58 and this reads as follows “*Among HIVST users, 893 (82.0%) returned to the clinic for confirmatory testing after testing negative on HIVST; 196 (17.9%) were confirmed HIV positive following a positive HIVST*”.
**Comment**

Line 66: For reasons given in #1 under general comments above, “feasible and” may be deleted.
**Response**

We appreciate the reviewer’s comment, we have changed the manuscript title as shown in line 1-2 and this reads as follows “*Acceptability of unsupervised peer-based distribution of HIV oral self-testing for the hard-to-reach in rural KwaZulu Natal, South Africa: Results from a demonstration study*”

**Comment**

Background:
Line 85: The sentence is not complete; something is missing before (1).
**Response**

We thank the reviewer for the comment, the missing number has been added as shown in line 94 and this reads as follows “Worldwide, 19% of PLHIV did not know their status in 2019 [2], while in South Africa this figure was 8%”

**Comment**
Line 85: There is an extra space between 2030- and stating
**Response**

We thank the reviewer for the comment and the extra space has been removed as shown in line 96.

**Comment**
Line 102: ‘services’ after (HTS) should be deleted
**Response**

We thanks the reviewer for the comment, services’ after (HTS) has been deleted as shown in line 110-111 and this reads as follows “*Studies have shown that HIVST can be a feasible option to improve uptake of HIV testing services (HTS) at clinics*”

**Comment**

Line 109: Change hyphen to comma in the ref (18-19)

**Response**

We thank the reviewer for the suggestion. Hyphen has been changed to comma and as shown in 127.

**Comment**

Line 112: I think, quite rightly include only ‘acceptability’

**Response**

We appreciate the reviewer’s comment.

**Comment**

Methods:
Line 166: Maybe in order to replace descriptive with cross-sectional analytical.

**Response**

We thank the reviewer for the comment. Descriptive has been changes to cross sectional analytical study. This as shown in line 134-138 and this reads as follows “*This was a cross-sectional analytical study of peer-led, unsupervised distribution model of HIVST kits, whereby, lay counsellors or community health workers provided HIVST kits to primary recipients (PRs) for distribution to their sexual partner, anyone in their social network (termed secondary recipients) or for self-testing.”*

**Comment**

Line 118: “areas’ – can one more be specific? -- like by administrative structure.

**Response**

We appreciate the reviewer’s comment. We changed areas to area shown in line 140 and this reads as follows “*Eshowe and Mbongolwane area in KwaZulu-Natal*”.

**Comment**

Line 133 (study definitions): It could be helpful to add here operational definitions for ‘acceptability’ and ‘hard to reach’.

**Response**

We appreciate the reviewer for the comment. Operational definition for the hard-to-reach has been added as shown in line 156-158 and this reads as follows “*Hard-to-reach groups to describe sub-groups of the population that may be difficult to reach through conventional HIV testing services (those who have no easy access to testing facilities) and those sub-groups could be men, adolescents and young adults*. ”. Furthermore, operational definition for “*acceptability” is found in line 170-172 and this reads as follows “Acceptability in this study was defined as the proportion of people invited to be PRs who agreed to take HIVST kits for use and distribute them to their sexual partner, family members or anyone in their social network*”.

**Comment**

Line 184: Need to add median to frequencies and proportions.

**Response**
We thank the reviewer for the comment. Data analysis section has been changed as shown in line 221-223 and this reads as follows “*Continuous variables were summarised using mean and standard deviation if normally distributed, otherwise median and interquartile range were used, and categorical variables were summarised using percentages or proportions.”*

**Comment**

Line 189 (Ethical considerations): Not sure, but wondering whether one would need to add a few lines addressing some key ethical issues here like consent and confidentiality, etc.
**Response**

We appreciate the reviewer for the comment. We have expanded ethical considerations section as shown in line 237-240 and this reads as follows “*PR and SR provided written informed consents before undergoing the study procedures and information such as age, gender, number of tests were collected. We did not collect any identifying information such as name, surname, address, or ID numbers.*”

**Comment**

Results:
Line 195: …. “PRs, of them 9,891(26.9%) accepted” may be edited as … 'PRs of which 9,891(26.9%) accepted.'
Table 1: Useful to add % men and % female for each group to show acceptability by sex.
**Response**

We appreciate the reviewer’s comment. We have addressed the comment as shown in line 243-245 and this reds as follows “*Between September 2018 and March 2020, 36,708 individuals were approached to participate in the study as PRs, of which 9,891(26.9%) accepted. Additionally, 2,973(30.0%) were men and 6,918(70.0%) were female.”*

**Comment**

Lines 207-209: This is a description of content Table 1, not in Table 2. Should move this to the description for Table 1.
**Response**

We thank the reviewer for the suggestion. The section was moved as suggested as shown in line 249-251 and this reads as follows “*Acceptability of HIVST was thrice as high at community-based testing sites than at PHC (64.5% vs. 21.0%; p=0.001). The median age was similar in both community-based testing sites and PHCs (p=0.389).*”

**Comment**

LINE 208: 64.5% here vs 63,5 in Table 1.
Table 2: Age group class intervals >25 to ≤40 and ≥40 overlap; please correct. All P-values should be in the variables’ value rows.
**Response**

We appreciate the reviewer for the comment. The inconsistency between number in the and the table has been fixed and this is shown in line 249-250 and it reads as follows “*Acceptability was thrice as high at community-based testing sites than at PHC (63.5% vs. 21.0%; p=0.001)*”. Additionally, we have corrected the age group class intervals >25 to ≤40 and ≥40 overlap as well as all P-values and these corrections are shown table 2 .

**Comment**

Line 223: '196 individuals or 17.9% (95% CI 15.7 to 20.3)' – Confidence interval is not shown in the figure or any of the table. One may edit this as “..196 (17.9%) of the individuals .”
**Response**

We thank the reviewer for the comment. This has been changed as shown in line 280 and reads as follows “*…196 (17.9%) of individuals were confirmed HIV positive*”.

**Comment**

Lines 221-224: Better to move this and include it in the description at the beginning of the section on 'Return for confirmatory testing and ART uptake' below, where Figure 2 =3 is presented.

**Response**
we thank the reviewer’s suggestion. We moved Figure 1 to align this with the text as shown 282-282.

**Comment**

Line 228: insert word median before 28.1 years.
**Response**

We appreciate the reviewer for the suggestion. We have inserted the word median as line 291 and this reads as follows “*The ages of PRs and SRs were similar, median age of 28.1 years (IQR: 23.5-34.7)…*”

**Comment**

Line 230. Remove the word 'furthermore' and just start the sentence with “Among”
**Response**

We appreciate the reviewer for the suggestion. The word ‘furthermore’ has been deleted as shown in line 301 and this reds as follows “*Among HIVST users, 12 (1.1%) reported never having been tested,…*”

**Comment**

Line 231: insert a comma after users.
**Response**

We thank the reviewer for the suggestion. Comma has been inserted as suggested as son inline 301 and tis reads as follows “*Among HIVST users, 12 (1.1%) reported never having been tested,…*”

**Comment**

Line 222-233: Remove 'In addition' and just start with 'Through'
**Response**

We thank the reviewer for the suggestion. We have removed ‘In addition’ as suggested , this is shown in line 302-303 and this reads as follows “*Through HIVST, HIV positivity was more than 10-fold higher in PHC than in community-based testing sites…*”

**Comment**
Table 3: Remove the full stop at the end of the title. Age group class intervals >25 to ≤40 and ≥40 overlap; please correct.
**Response**

We appreciate the reviewer’s comment. Comma has been removed from the tile as show in table 3.

**Comment**
Line 241. Add 'a median time of' before 'two months' and add (100%) after 190 and Line 242. Insert space between 36 and (18.4%).
**Response**

We appreciate the reviewer for the suggestions. We have added these as shown in line 309-311 and these read as follow “…*all reported having performed the HIVST within a median time two months (IQR: 1.15-2.85). Of 196 (100%) confirmed HIV positive through HTS, clinical records of 36 (18.4%) participants could not be found after extensive* search of electronic registers for the study sites (**Figure 1**).*”*

**Comment**

Line 247: Delete 'Linkage to care and ART initiation'
**Response**

We appreciate the reviewer’s comment, this has been deleted.

**Comment**
Discussion:
Line 256: May need to delete 'and feasible' for reasons given above.
**Response**

We appreciate the reviewer’s comment . the word ‘feasible’ has been deleted as shown in line 321-322 and this reads as follows “*Distribution of HIVST kits to persons attending primary care clinics or community-based testing sites for self-testing and/or distribution to peer was acceptable*”.

**Comment**
Line 263: SSA expanded here; the acronym should be used subsequently (lines 280, 385) – please edit.
**Response**

We thank the reviewer for the comment. The acronym has been used as suggested, this is shown line 358-361 and it reads as follows ‘*Linkage to confirmatory testing and HIV care after a positive HIVST has been highlighted as a major concern in literature from SSA given that not many people have the motivation to seek confirmatory HIV testing and/or linkage to care following an HIV- positive self-tested result’*

**Comment**
Line 267: Replace 'This' with 'Our', otherwise one may This to mean the studies describe in the preceding sentence.
**Response**

We thank the reviewer for the comment. we have replaced ‘This’ with ‘Our’ as shown in line 333 and this reads as follows “Our finding suggests making HIVST more accessible..”

**Comment**
Line 271: Edit '1089' as '1,089'

**Response**

We appreciate the reviewer’s suggestion. This has been edited accordingly as suggested as shown in line 337-338 and it read as follows “*During the study period, 1,089 individuals reported HIVST use; of these 58.8% tested themselves*”.

**Comment**
Line 330 (Conclusion): there is more in the conclusion of the Abstract than here. May need to add some more here.

**Response**
We thank the reviewer for the comment. We have added a sentence in the conclusion as shown in line 432-435 and this reads as follows “*Acceptability to be PRs was thrice as high in community sites compared to clinics. However, few of those secondary recipients who reported were first time testers, young adults, and men, indicating this distribution model did not reach the hard-to-reach population.”*

**Comment**

Acknowledgment:

Line 343: Add 'and' between HIVST, and conducted

**Response**

We appreciate the reviewer’s comment and this has been addressed accordingly as shown in line 449.

**Comment**

Availability of data and materials
Please see the Journal guidelines where to include this.

**Response**
We thank the reviewer for the comment, we have moved the section accordingly and this shown in line 441-443.

# Reviewer #3

This paper presents a model to distribute oral HIV self-tests (HIVST) in rural South Africa and describes the acceptability and feasibility of unsupervised peer distribution of HIV self-testing. This paper offers an a potentially important model for reaching hard to reach rural populations with HIV testing; however, there are a number of issues with the paper in its current form that warrant further attention. In particular:

**Comment**
The description of previous studies of HIVST in lines 110-113 is not clear. I suggest the authors state more clearly what these previous studies found and how the current study adds to this literature.

**Response**

We appreciate the reviewer’s comment. We have rephrased the paragraph as shown in line 110-114 and this reads as follows “*Evidence from South Africa has demonstrated that HIVST can be a feasible option to improve uptake of HIV testing services (HTS) at clinics, and the use of HIVST in an unsupervised environment can be feasible and the results so obtained can be reliable However, linkage to care and confirmatory testing for the unsupervised HIVST and further care were relatively low*”.

**Comment**
The “Design” section of the Methods (lines 115-116) does not describe the evaluation design of this study.

**Response**
We thank the reviewer for the comment. We have expanded the study design section and shown in line 134-138 and this reads as follows “*This was a cross-sectional analytical study of peer-led, unsupervised distribution model of HIVST kits, whereby, lay counsellors or community health workers provided HIVST kits to primary recipients (PRs) for distribution to their sexual partner, anyone in their social network (termed secondary recipients) or for self-testing. In addition, data was collected prospectively among those returned for confirmatory testing*”.

**Comment**

The “Study definitions” section (lines 133-139) seems like it would be better labelled “participants”.

**Response**

We appreciate the reviewer’s comment. We added additional operational definitions to this section unrelated to the study participant and this is shown in line 160-172. Therefore, we maintain the current wording.

**Comment**
The authors note in lines 148-149 that persons diagnosed with HIV were excluded from participation. Can the authors explain why that was, given that one of the purposes of distributing HIVST kits is to offer sexual partners an opportunity to test?
**Response**

We thank the reviewer for the comment. We fully agree with reviewer assertion that excluding those whom tested positive may have missed an opportunity to get their partners tested. However, excluding patients newly diagnosed with HIV on the day of enrolment was motivated by the fact predominant majority of newly diagnosed patients are not always in right state of mind considering the adverse psychological burden they present as they just discovered to have life changing disease. Asking these patients to consent in order to take part in the study that there is direct benefit would spare unnecessary emotional and psychological distress. Due to these reasons we decided to excluded them in the study.

However, we have added an explanation as shown in line 183-184 and this reads as follows *“Those diagnosed with HIV on the day of visit were excluded given the emotional and psychological distress that these usually present as they just discover to have a life changing disease.”*

**Comment**

In the “Data analysis” section (lines 182-184), the authors define “acceptability” as “the proportion of people invited to be PRs who accepted HIVST”. This is a bit confusing to me – is acceptability the number of people who agreed to take the tests and distribute them to others or is it the number of people who agreed to use the self-test?

**Response**

We thank the reviewer for the comment and suggestion. We have addressed definition as per reviewer suggestion, this is shown in line 170-172 and this reads as follows *“HIVST kits to their sexual partner, family members or anyone in their social network”.*

**Comment**

Acceptability is typically defined as uptake – that is – are people willing to use the test. It has been pretty well established in the literature (including what is cited in this paper) that HIV self-testing is acceptable to most people in South Africa. I think the potential contribution of this paper is on the effectiveness (or not) of the distribution model for hard-to-reach populations in rural settings. That is, did it improve testing access or coverage in community and/or clinics settings? What evidence do the authors have to show that and how did it improve access and/or coverage specifically within the clinics (or were the kits taken outside of the clinic for broader distribution?) And beyond the community testing site? More on the how would be helpful.

**Response**

We thank the reviewer for the comment and for the reflection around HIV self-testing in South Africa. We acknowledge the reviewer comment on the literature landscape of HIV self-testing in South Africa, among other higher of acceptability of HIV self-testing in South Africa. However, there is limited evidence on the acceptability of unsupervised peer-distribution of HIV self-testing under programmatic conditions, particularly in HIV-high prevalence and low-income rural settings. Therefore, this paper addressed this research gap. Furthermore, his paper is of particular significance since it is a demonstration study assessing an acceptability of unsupervised peer-distribution of HIV self-tests or peer-led distribution as method to scale-up HIV testing in general population, under programmatic conditions rather than targeted interventions (FSWs, post-partum and pregnant women, fishing community, and MSM).

This paper further assessed ART initiation for participants who tested HIV-positive using HIVST. This paper highlights evidence that making HIVST more accessible at community-based testing sites might help to improve testing uptake and HIV diagnosis in Sub-Saharan Africa, especially in those who test frequently.

**Comment**
In Table 1 and in the description of the data (lines 195-201), it seems that the authors are describing acceptability as the number of people who were willing to take the HIVST kits and distribute them – is this correct? Also, the authors report on acceptability again in lines 207-208 showing a difference between community and primary health clinic sites. This is a bit confusing as most people think of this as how many people actually took the HIVST kit to test/use rather than the distributors. Is there a way to label this differently so it is clearer to the reader what the authors are describing and why that is important (e.g., the characteristics of the distributors?) or is this describing the characteristics of the people who actually tested with the HIVST kits?

**Response**

We appreciate the reviewer’s comment. Under recruitment section , line 178-181 we stated as follows “*Individuals who expressed interest were given detailed information about the study and screened for eligibility.After informed consent, they were enrolled in the study as PRs and given between 2-5 HIVST kits, based on their choice, for distribution and/or self-testing.”* We further stated in line 188-191 as follows “*PRs were instructed to distribute HIVST kits to their sexual partner, family members or anyone in their social network who was above 18 years old. They could also test themselves if they were HIV-negative.”* These two statement highlight that once an individual was enrolled in the study as primary recipients would have not only distributed HIVST kits but would have also tested themselves if they were HIV-negative.

Furthermore, to avoid confusion we have rephrased and moved the following (previously line 207-208) “*Acceptability of HIVST was thrice as high at community-based testing sites than at PHC (63.5% vs. 21.0%; p=0.001). The median age was similar in both community-based testing sites and PHCs (p=0.389).”* to “*Acceptability was thrice as high at community-based testing sites than at PHC (63.5% vs. 21.0%; p=0.001)*” this is shown in line 249-251.

**Comment**

The authors report in lines 221-227 that of 34,715 adults that were tested for HIV at both primary care clinics and community-based testing sites during the study period, only 1,089 (3%) reported testing with an HIVST. This number is quite low and suggests potentially low coverage of distribution of the >31,000 HIVST kits or potential low uptake/use of the kits given the prevalence in the region and undiagnosed persons with HIV. The authors should consider why the number overall is low and discuss potential reasons for this in the Discussion section.

**Response**

We thank the reviewer for this comment and we concur with the reviewer that only 1,089 out 34,715 or 3.1% of individuals reported having used HIVST and this could mean low coverage distribution of the 31,341 in the study setting. We have acknowledged the low coverage distribution as one many study limitations as shown in line 409-412 and this reads as follows “*An inherent limitation of many studies of unassisted self-testing is the difficulty of verifying use and accuracy of the tests. In our study all information in the follow-up component was obtained through participants’ reports, and thus we were unable to verify use, or results.”*

Additionally, the low uptake of HIVST does not necessarily translate in higher proportion of undiagnosed considering our study setting is characterized by a high HIV testing coverage, population cross sectional survey showed that 96.7% of the individuals surveyed reported having at least one HIV test in the survey^[1]^.

**Comment**
• In this same section, the authors report that the majority of the reported users of the HIVST kits were the distributors (primary recipients – 59%) who had mostly been tested within the past 6 months. This begs the question then of how this model is improving access to testing and coverage since most of these folks may likely be retesting.

**Response**

We appreciate the reviewer’s comment. We fully agree with the reviewer’s comment. This study was not a surprising result considering the high HIV testing coverage in the study setting (see previous response), however, we have is extensively argued on this as shown in line 337-357 and this reads as follows “*During the study period, 1,089 individuals reported HIVST use; of these 58.8% tested themselves. Thus, not only did people agree to distribute HIVST, but they also used it as a method to test themselves in private. This can help, along with reaching more people, also to increase HIV testing frequency. Furthermore, a higher proportion of HIVST users had tested less six months before and a smaller proportion were first-time testers. This is not surprising considering the high HIV testing coverage in the study area [22]. Further research is needed on how HIVST will co-exist within HTS and on the role of HIVST in settings with high HIV testing coverage.”*

**Comment**
• The data in Figure 1 are a bit misleading. Specifically, in the column on the left it seems that the 36 confirmed positive persons whose records could not be found should not be removed from the denominator in the last box as these were confirmed positives but did not link or initiate ART. Similarly, the 25 already on ART seem to belong in the denominator as well since they are on ART. If the 25 are added back in, the number initiated or on ART would be 154/196 confirmed positives (79%) with 36 having no record of initiating ART and 6? This is a more accurate estimate of the number of PLHIV who initiated or were on treatment among the 196 HIVST PLHIV identified. The current estimate is an overestimate.

**Response**

We appreciate the reviewer’s comment. We have added 36 confirmed HIV positive whose records could not be found in the denominator, this is shown Figure 1. However, 25 confirmed HIV positive patients who were already on ART before receiving HIVST could not be added in the denominator. We have provided reasons these patients could not be added in the denominator, this is shown in line 310-319 and this reads as follows “*Of 196 (100%) confirmed HIV positive through HTS, clinical records of 36 (18.4%) participants could not be found after extensive search of electronic registers for the study sites (****Figure 1****). These were categorized as not linked to care. In addition, 25/160 (15.6%) were already on ART before receiving HIVST with median time in care of 53.2 months (IQR: 32.8-68.6), 129/171 (75.4%) initiated ART, whereas 2,362/2685 (88%) of HIV positive HIVST non-users-initiated ART.”*

Additionally, we have comprehensively argued in the discussion section about those HIV-positive re-confirmed their HIV status using HIVST in line 376-383 and this reads as follows “*Over 10% of those confirmed HIV positives had been diagnosed prior to study enrolment, despite clear instruction that the test should not be used by PLHIV on ART and waning of false positive HIVST if on ART [6, 29]. Repeat testing by those on ART has been reported elsewhere [28, 30] and suggests that PLHIV desire to re-confirm their HIV status for themselves, despite their reluctance to disclose their status. Programs should spend more time on patient education so that those on ART must no use HIVST.* ”

**Comment**
As shown above, the HIVST users actually had lower treatment initiation or on ART rates than those tested through other methods. How to address this issue and strengthen linkage to care for HIVSTers should be addressed in the Discussion section.

**Response**

We appreciate the reviewer’s comment. We have written a paragraph relative to ART initiation among HIV user as shown in line 384-398 and this reads as follows “*An important contribution of this study was an attempt to establish the proportion of individuals who returned for confirmatory testing and ART uptake following HIVST. In our study, confirmatory testing and ART uptake was lower among HIVST users compared to HIVST non-user, 75.4% and 88.0% respectively. The high proportion of ART initiation among HIV non-users could be because this study was conducted in a setting where UNAIDS 90-90-90 targets were achieved in 2018 [22] and in era of universal test and treat (UTT). UTT interventions are intensive testing programmes designed to find even the hardest-to-reach populations. Systematic review and meta-analysis of linkage to treatment or care following HIVST in the general population has found limited but encouraging evidence of successful linkage among HIVST users in the general population [31]. Further research is needed to assess cost-effective and sustainable interventions designed to strengthen linkage to treatment and care following HIVST, such as in person referral and home ART initiation. Additional data is needed to estimate the effect on HIVST on continuity of care or viral suppression outcomes as well as linkage to prevention services among those who are HIV-negative and at ongoing risk under programmatic conditions, particularly in HIV-high prevalence and low-income rural settings*.”

**Comment**
The Discussion section often repeats the results. Suggest that the authors briefly summarize the key findings without restating the data and offer information to help interpret the finding or discuss how it supports or contradicts the extant literature.

**Response**

We thank the reviewer for the comment. we have repeated some of the results throughout the discussion section.

**Comment**
Overall, this study has potential to propose a model for distribution of HIVST kits for use by hard-to-reach populations in rural settings in South Africa. However, there are current limitations in the way the data is presented and the lack of clarity of the findings that limit the interpretation of the data and its current contribution to the literature. The authors are encouraged to present the data in a way that would clearly show who is being reached with these test kits and if they are truly increasing access to testing in these populations (especially men and young people which appear to account for about a third of those using HIVST kits) or if they are, for example, primarily providing retesting options for those who are already testing regularly every 6 months.
**Response**

We thank the reviewer for the comment. The reviewer remarks have been taken into consideration while addressing his comments. We would like to emphasise that the main objective of this study was to describe acceptability of unsupervised peer-distribution of HIVST as method to scale-up HIV testing in the general population, particularly under programmatic conditions. Considering innovative delivery models of HIVST provide an opportunity to reach the hard-to-reach population (young men and those never tested). The study has shown that 26.9% of people approached to be peer-distributor or primary recipients accepted. However, the distribution model could not reach the hard-to-reach such men , young adults , and first time testers as anticipated.

We have made several adjustments throughout the manuscript to reflect your comment. For instance, 432-435 acknowledge failure of this distribution model to not reach the hard-to-reach. However, surprisingly results were that taking HIVST kits outside of the conventional health facilities resulted in broader distribution and high acceptability at community-based testing sites, see line 403-406 and 432-435.

**Comment**

The paper needs a copyediting review before it is re-submitted as there are a number of missing words, spaces, and punctuation.

**Response**

We appreciate the reviewer’s comment. The paper has undergone major editing.

Once again, we would like to thank the reviewers for the valuable comments and inputs that have helped to improve the quality of our Manuscript.

We trust you will find the revised document and the responses in order.

Yours Sincerely

Marcel Kanyinda Kitenge

1. Médecins Sans Frontières. Mbongolwane and Eshowe HIV Impact in Population survey
